# Successful treatment of thromboses of major arteries after ChAdOx1 nCov-19 vaccination

**DOI:** 10.1186/s42466-021-00151-y

**Published:** 2021-10-04

**Authors:** Yasemin Goereci, Nina N. Kleineberg, Marie Madlener, Hannah Neuschmelting, Gereon R. Fink, Clemens Warnke, Henning Stetefeld

**Affiliations:** 1 Department of Neurology, Faculty of Medicine and University Hospital Cologne, University of Cologne, Cologne, Germany; 2 Cognitive Neuroscience, Institute of Neuroscience and Medicine (INM-3), Research Centre Jülich, Jülich, Germany; 3 Department of Radiology, Faculty of Medicine and University Hospital Cologne, University of Cologne, Cologne, Germany

## Abstract

The ChAdOx1 nCoV-19 adenoviral vector vaccine to prevent contracting Covid-19 caused by infection with SARS-CoV-2 has been associated with vaccine-induced immune thrombotic thrombocytopenia (VITT) primarily leading to venous thromboses. Here, we report two cases of major arterial occlusions after ChAdOx1 nCov-19 vaccination, comprising a 42-year-old woman with thrombotic occlusion of the left carotid artery, and a 62-year-old man with occlusion of distal aorta and iliac arteries. Both were successfully treated with intravenous immunoglobulins and non-heparin anticoagulant agents leading to a beneficial short-term outcome of 6 weeks in case 1 and four months in case 2.

Our first case, a 42-year-old female without pre-existing conditions developed visual impairment and severe headache nine days after the first dose of ChAdOx1 nCoV-19 vaccine. Two days later, she presented with transient hemiparesis and aphasia lasting ten minutes. Platelet counts were low (nadir 40.000/mm^3^) and D-dimer levels markedly increased (35 mg/l). Cerebral magnetic resonance imaging (MRI) revealed thrombotic occlusion of the left carotid artery, commencing at the bifurcation with resulting ischemia in the territory of the middle cerebral artery (Fig. 1).

**Fig. 1:**
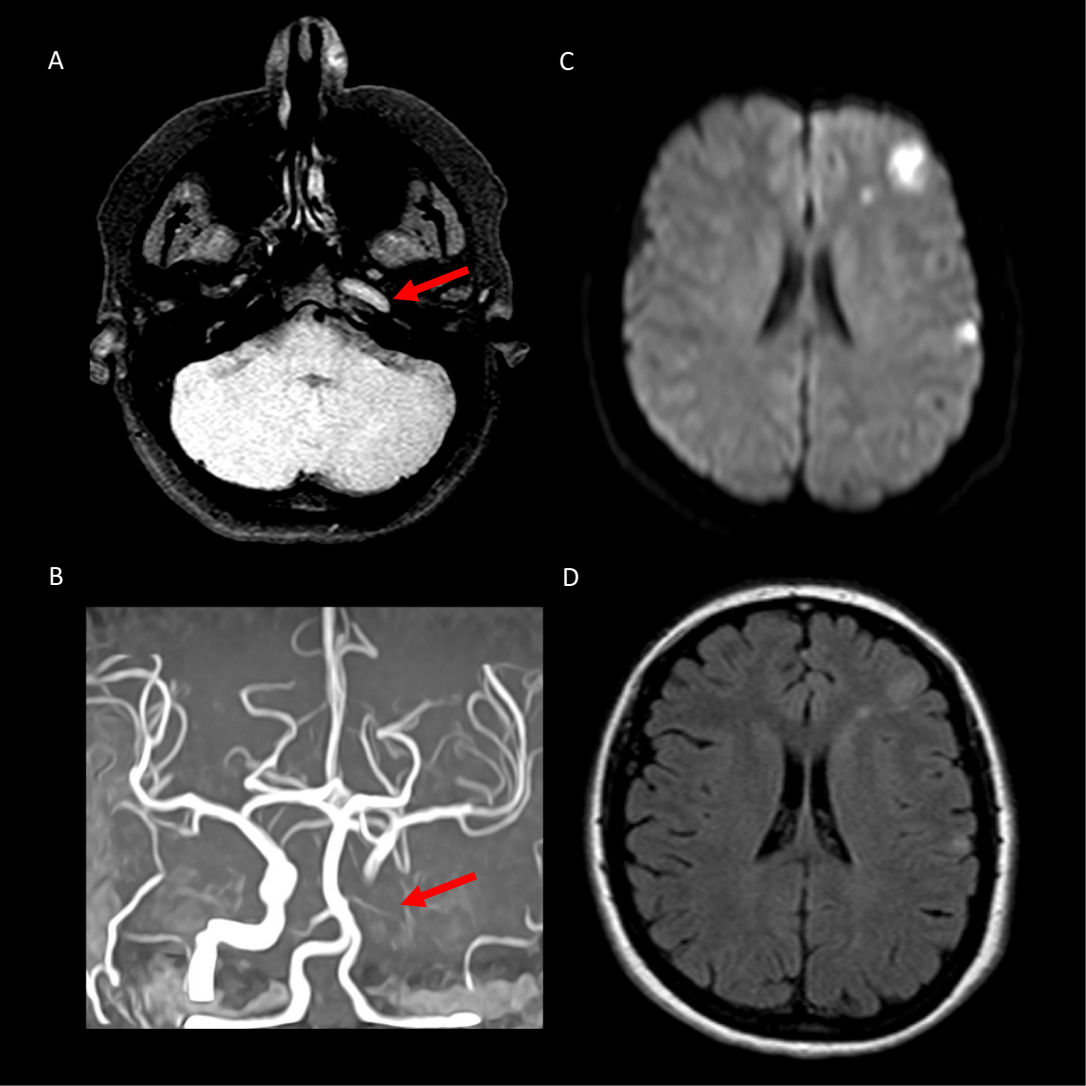
**a** Axial T1 fat saturated and **b** coronal Time Of Flight (TOF) MRI images at 1.5 T show evidence of left intracranial ICA thrombotic occlusion in petrous to clinoid ICA segments. **c** Axial diffusion weighted (DW) and **d** Fluid Attenuated Inversion Recovery (FLAIR) images reveal concomitant downstream infarction in MCA territory

Secondly, a 62-year-old male with hypertension treated with bisoprolol developed acute, painful and pulseless lower limb paraparesis thirteen days after the first dose of ChAdOx1 nCov-19 vaccination. Thrombocytopenia (nadir 53.000/mm^3^), and elevated D-dimer levels (2.8 mg/l) were detected. Cerebral and spinal MRI showed no signs of acute ischemia. However, ultrasound studies demonstrated reduced blood flow in the common iliac arteries with an undetectable left dorsalis pedis artery. Computed tomography angiogram (CT-A) confirmed thrombotic occlusion of the distal aorta below the renal arteries reaching into both common iliac arteries as well as segmental lung artery embolism (Fig. 2). Immediate surgical aortal thrombectomy and fasciotomy of the lower limbs was conducted to prevent compartment syndrome.

**Fig. 2:**
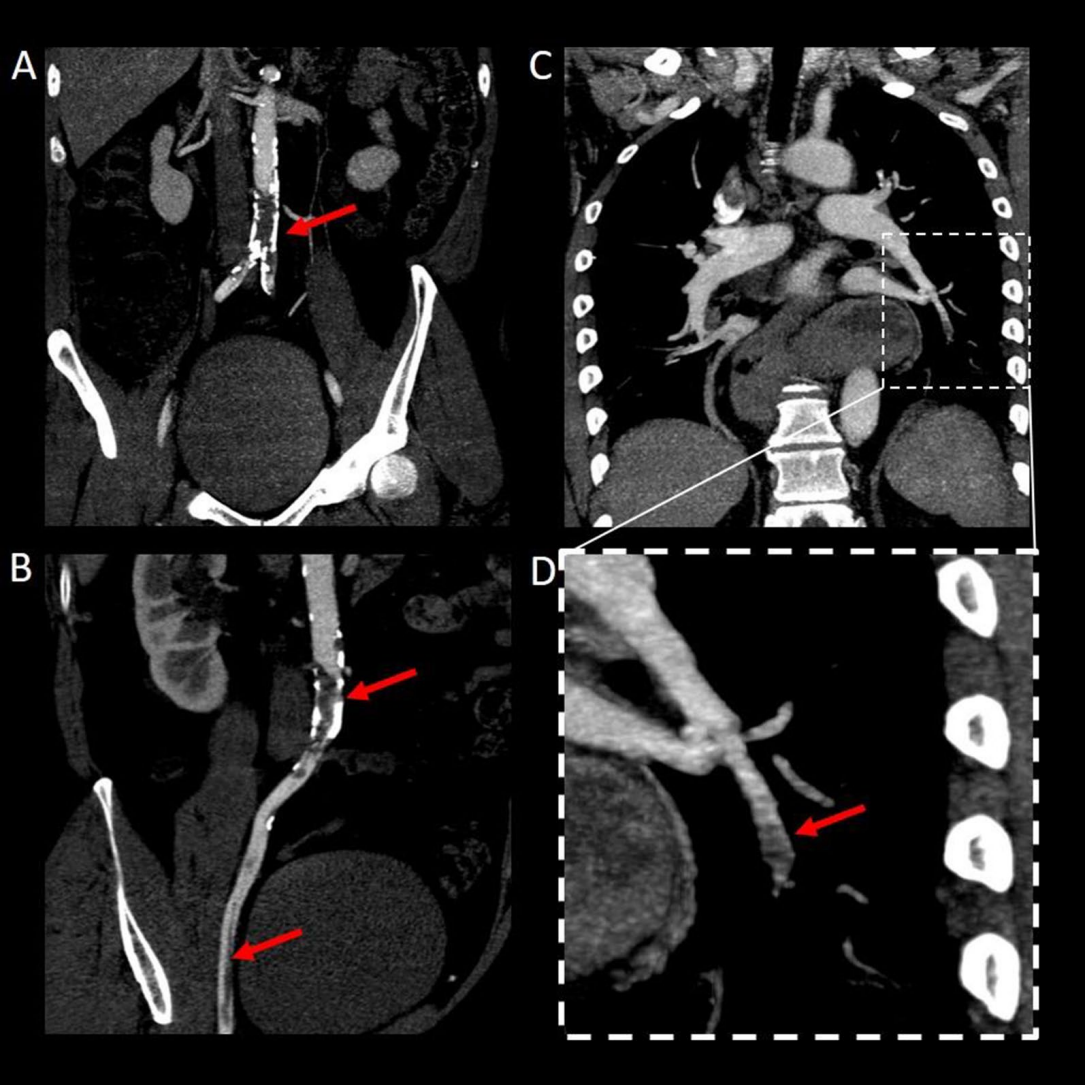
**a** Coronal and **b** curved multiplanar reconstructions of CT-A (in 5 mm maximum intensity projection) display subtotal thrombotic occlusion of infrarenal aorta and extension of thrombus into both common iliac arteries and right external iliac artery. **c** Coronal multiplanar reconstruction of CT-A (5 mm maximum intensity projection) and **d** magnified image show segmental pulmonary artery embolism

No prior coagulopathy or heparin exposure was present, and both patients displayed thrombocytopenia and atypical arterial thromboses, developed within two weeks after ChAdOx1 nCov-19 vaccination. Platelet factor 4 (PF-4) autoantibodies were assessed with enzyme-linked immunosorbent assay and the PF4-enhanced platelet activation test, confirming the diagnosis of vaccine-induced immune thrombotic thrombocytopenia (VITT) [1, 2]. Other causes of thrombocytopenia, i.e., heparin-induced thrombocytopenia, idiopathic thrombocytopenic purpura, antiphospholipid syndrome, thrombotic thrombocytopenic purpura, atypical hemolytic uremic syndrome and idiopathic thrombocytopenia were ruled out. Diagnostics including 72-h cardiac monitoring, transesophageal ultrasound, and duplex sonography of carotid arteries did not reveal other etiologies of thromboembolism. Both patients were treated with intravenous immunoglobulins (IVIG, 1 g/kgBW/d for two days) and argatroban (targeting factor 2 of the initial activated partial thromboplastin time) following current expert-opinion recommendations [1]. Platelet counts stabilized and no recurrent thromboses occurred.

The first patient was discharged after 15 days with no neurological deficit, normal platelet counts, and completely recanalized carotid artery on ultrasound at day 10. Argatroban had been replaced by apixaban (2 × 5 mg) prior to discharge. Due to recurrent thrombocytopenia (nadir 90.000/mm^3^, day 29), treatment with IVIGs (1 g/kgBW/d for two days) was repeated. Oral anticoagulation was continued up to the last follow-up at month 4 without recurrent thromboses.

In the second patient, post-surgical creatine kinase peaked at 19.512 U/L, and was remittent with infusion therapy averting the risk of kidney failure. He recovered well from fasciotomy with a vacuum assisted closure-therapy. The pulmonary artery embolism remained asymptomatic. Argatroban was administered until day 14, then substituted by rivaroxaban (2 × 15 mg) continued until day 21. Further, dosage of 1 × 20 mg was planned for the next 3 months, to be reassessed later on. A mild paraparesis and moderate dysesthesia persisted at discharge to a rehabilitation center 24 days after admission, presumably caused by the leg ischemia as a spinal ischemia was not detectable also on a follow-up MRI.

The current German recommendations^1^ suggest the use of the ChAdOx1 nCoV-19 vaccine in persons above the age of 60, arguing that, in particular, women < 60 years are predisposed for thromboses, and that VITT was predominantly noted in patients below 60 years as described in earlier studies [1–3]. Our cases illustrate that VITT causes not only venous thrombosis but also—albeit less frequently—cerebral as well as non-cerebral arterial thromboembolism, and male individuals beyond the age of 60 years can be affected as well [4–6]. This is in concordance with a recent study^2^ and previous case reports [4]. So far, one case with a possible VITT after the RNA–1273 vaccine [7] has been reported, besides multiple cases with the vector based ChAdOx1 nCov-19 as well as Ad26.COV.2.S vaccines [8].

Early consideration of VITT and rapid diagnosis with an approved PF4-ELISA and targeted therapy with immunoglobulins plus infusion of non-heparin anticoagulant agents are pivotal to avoid unfavorable outcome and to decrease VITT-associated mortality [4]. Possibly, the incidence of VITT following the ChAdOx1 nCoV-19 vaccine might be underreported as no routine coagulation diagnostic is performed in vaccinated patients without (symptomatic) thromboembolic events [4]. Due to the suspected pathogenesis, transfusion with platelets should be avoided to hinder aggravation of thrombotic incidents [3].

Since no long-term data subsequent to VITT is available yet, follow-ups with bloodwork and clinical exams are advisable. Notably, the first patient presented recurrent thrombocytopenia after initial normalization of platelet counts. Thus, to date, duration of VITT and its continued implications on coagulation remain unclear until more clinical data becomes available. Repeated intravenous IVIGs therapy might stabilize thrombocytopenia and prevent reoccurring thromboses.

The presence of thromboses, thrombocytopenia, highly elevated D-dimer levels, and lowered or normal fibrinogen level seem to be characteristic for the syndrome with an onset of 5–24 days after ChAdOx1 nCov-19 vaccination [3, 4].

## Data Availability

Available at request.

## Footnotes

1 Stellungnahme der Ständigen Impfkommission zum Zeitpunkt der Gabe eines mRNA-Impfstoffs nach Erstimpfung mit AstraZeneca Vaccine (Vaxzevria) bei < 60-Jährigen (https://www.rki.de/DE/Content/Kommissionen/STIKO/Empfehlungen/Stellungnahme-Impfabstand.html), from 14th April 2021, searched on 09th May 2021.
